# Methionine Antagonizes Liver and Kidney Antioxidant Function Damage in Heat-Stressed Rex Rabbits

**DOI:** 10.3390/ani15081148

**Published:** 2025-04-16

**Authors:** Shu Li, Xiaosong Wang, Gongyan Liu, Lei Liu, Fuchang Li

**Affiliations:** 1 Key Laboratory of Efficient Utilization of Non-grain Feed Resources (Co-Construction by Ministry and Province), Ministry of Agriculture and Rural Affairs, Shandong Provincial Key Laboratory of Animal Nutrition and Efficient Feeding, College of Animal Science and Technology, Shandong Agricultural University, Taian 271017, China; 2021010083@sdau.edu.cn (S.L.); 14768123432@163.com (X.W.); 2 Shandong Academy of Agricultural Sciences Institute of Animal Husbandry and Veterinary Medicine, Jinan 250100, China; gongyanliu@foxmail.com

**Keywords:** methionine, heat stress, oxidative stress, Rex rabbit, Nrf2 pathway

## Abstract

Heat stress triggers systemic oxidative stress that compromises physiological homeostasis. This study evaluated methionine’s effects on hepatic and renal antioxidant capacity in heat-stressed Rex rabbits. Rabbits were divided into five groups (30 replicates/group): control (20–25 °C, basal diet), heat stress (HS, 30–34 °C, basal diet), and HS +0.15%, 0.3%, or 0.45% methionine-supplemented groups. After 21 days, serum, skin, liver, and kidney samples were analyzed for biochemical parameters, oxidative stress markers, and gene expression. Results showed that 0.15–0.3% methionine supplementation under heat stress increased methionine apparent digestibility and suppressed amino acid catabolism; decreased serum alanine aminotransferase (ALT) and aspartate aminotransferase (AST) levels; reduced skin malondialdehyde (MDA) and elevated (MSRA) activity; attenuated hepatic central venous congestion and renal tubular vacuolization; enhanced hepatic superoxide dismutase (SOD), catalase (CAT), and glutathione peroxidase (GPx) activities (0.3% group); and modulated antioxidant gene expression via Nrf2/HO-1 and Nrf2/NQO1 pathways. Pathological analysis confirmed reduced fibrosis and cellular damage in liver/kidney tissues. Optimal methionine supplementation (0.3%) effectively mitigated heat-induced oxidative organ damage by enhancing endogenous antioxidant defenses and regulating redox-sensitive signaling pathways. These findings provide a nutritional strategy for alleviating heat stress-related metabolic disorders in rabbits.

## Simple Summary

Global climate warming has intensified extreme heat events, challenging intensive Rex rabbit farming. As economically valuable fur-meat hybrids, Rex rabbits are highly susceptible to heat stress due to underdeveloped sweat glands. This study explored methionine as a safe, eco-friendly dietary additive to counteract heat-induced damage. Using a heat stress model, we demonstrated that methionine supplementation effectively alleviated hepatic and renal oxidative injuries in heat-stressed rabbits. These findings propose methionine as a viable nutritional strategy to enhance thermotolerance in commercial rabbit production under escalating climatic stressors.

## 1. Introduction

Global warming has intensified environmental stressors, particularly heat stress (HS), emerging as a critical threat to livestock productivity and welfare [1]. Among agricultural species, rabbit farming holds unique advantages. FAOSTAT agricultural data highlighted stable exported quantity and price of rabbit meat from China in the past decade, driven by its low carbon footprint, manageable experimental scale, and dual-purpose utility for high-quality fur and lean meat production [2]. However, Rex rabbits exhibit marked thermal vulnerability due to anatomical constraints: absence of functional sweat glands, limited thermoregulatory capacity, and dense fur insulation that impedes heat dissipation [3]. While evolutionary adaptations, including seasonal hair shortening, skin thinning, and reduced follicular density, have partially mitigated thermal load, chronic HS persistently disrupts redox equilibrium, suppresses immune function, and induces multi-organ pathology—manifestations that ultimately impair growth and survival [4,5]. As metabolic hubs, the liver and kidney are primary HS targets [6]. During HS, the liver orchestrates energy mobilization and accelerates protein and carbohydrate catabolism to meet heightened metabolic demands, which could deplete growth-critical nutrient reserves [7,8]. Compromised hepatic recovery post–HS exposure correlates with MDA-mediated inflammatory cascades, wherein lipid peroxidation products perpetuate ROS-MDA feedback loops, exacerbating oxidative tissue damage [9,10]. Concurrently, renal dysfunction arises from HS-induced hemodynamic alterations (reduced glomerular filtration rates and tubular electrolyte imbalance), synergistically promoting oxidative stress and microcirculatory impairment in nephrons [11].

Current HS mitigation strategies encompass environmental modification, genetic selection, and nutritional intervention, with the latter offering immediate practicality in intensive farming systems [12]. As an essential amino acid, methionine (Met) plays critical roles in biological systems. Met deficiency disrupts protein biosynthesis and compromises organismal integrity, particularly during wound healing. Through multiple molecular mechanisms, Met demonstrates protective effects against membrane lipid peroxidation and oxygen-free radical-induced organelle damage [13]. Notably, under stress conditions, methionine residues adjacent to phosphorylation sites undergo preferential oxidative modification in biological systems [14]. This amino acid functions as an endogenous antioxidant through dual protective mechanisms: a repair system to reverse oxidative modifications and a clearance system to eliminate irreversibly damaged macromolecules [15]. Furthermore, Met exerts antioxidant properties via the MSRA pathway. MSRA overexpression significantly enhances cellular antioxidant capacity [16]. Dietary Met supplementation effectively maintains physiological homeostasis and strengthens cellular defense mechanisms against environmental stressors, including thermal challenges, while excessive methionine can cause oxidative stress [17]. Recent studies demonstrated that selenmethionine can alleviate intestinal oxidative stress and liver injury induced by aflatoxin [18].

The current research identifies two principal signaling pathways involved in HS-mediated immune dysfunction in livestock: the NF-κB pathway and the HSP (heat shock protein)-regulated stress response pathway [19]. Nuclear factor erythroid-related factor 2 (Nrf-2) is a pivotal regulator of cellular REDOX homeostasis. Activation of the Nrf2-Keap1 pathway boosts antioxidant response element-associated molecules. It governs the motilities of antioxidant enzymes such as SOD (the first defense against superoxide free radicals), GPX (a secondary auspice against hydroperoxide), and CAT to secure cells from oxidative damage [20,21].

Despite established correlations between HS-induced organ damage and oxidative biomarkers, the tissue-specific protective mechanisms of methionine in heat-stressed Rex rabbits remain underexplored. This research aimed to investigate methionine’s impact and latent mechanism on liver and kidney functions and oxidative stress in rabbits experiencing heat stress.

## 2. Materials and Methods

### 2.1. Animals and Experimental Design

A total of 150 Rex rabbits aged 2 and a half months with similar body weight (1949 ± 21 g), half of them male and half female (the sex of the Rex rabbits did not affect the test results), were randomly divided into five groups: control group (basal diet, 22–26 °C), heat stress experimental group (basal diet, 30–34 °C), and heat stress Met groups (basal diet+ 0.15%, 0.3%, 0.45% Met, 30–34 °C). Each group had 30 replicates, with one rabbit per replicate [22]. Single cage, single animal, raised in a self-made cage of 60 cm × 40 cm × 49 cm, following the natural light cycle and with no antibiotics used during the experiment. The intelligent automatic temperature control heating system was used to maintain the temperature of the rabbit house at 22–26 °C in the constant temperature control group and 30–34 °C in the heat stress group, and the humidity was recorded at 32–66%. The pre-test period was 3 d, and the test period was 21 d. According to the formula of Marai [23], the temperature and humidity index of the control group was less than 28, indicating a state of no heat stress, and heat stress time of the experimental group was 9:30–18:00 during the experiment. Three days before the experiment ended, 8 rabbits from each group were randomly selected according to average body weight and placed into metabolic cages under their respective experimental conditions for metabolic tests. The free intake of food, adequate water, and ventilation were ensured. Fecal samples were collected at the same time every day as feed samples. The formal trial commenced on 8 August 2020 and concluded on 28 August 2020 (China Standard Time). Experimental Rex rabbits were purchased from the Taishan rabbit farm. The experiment was conducted in the Rabbit Precision Farming Laboratory (National ISO17025 laboratory accreditation system) of Shandong Agricultural University (117°9′ E, 36°9′ N). All procedures involved in this study were approved by the Shandong Agricultural University Experimental Animals Ethical Review Committee (SDAU20180905). The content of Met in basal diet was 0.19% (Table A1).

### 2.2. Organ Index and Histopathological Observation

Upon the experiment’s termination, the rabbits within the metabolic cage were sacrificed (the rabbits were electroshocked by qualified people and slaughtered quickly with sharp knives), the surface fat, fascia, and blood were removed, and the liver and kidney were weighed. The organ index was computed as follows: organ index (g/kg) = organ weight (g)/live weight before slaughter (kg). Subsequently, skin tissues were frozen, and two parts of liver and kidney tissues were taken—one part was frozen, and the other part was fixed with 4% paraformaldehyde for 4 weeks (with fresh 4% paraformaldehyde being replaced every 7 days), followed by dehydration using an alcohol gradient and embedding in paraffin. Sections were sliced on an automatic rotary paraffin microtome (HM355S, Shanghai, China) and stained with hematoxylin and eosin. Histopathological alterations were observed via the Nikon microscope NIS-Elements D4.20.00 system.

### 2.3. Quantifying Methionine Concentrations Using the LC-MS External Standard Method

The LC-MS external standard approach was used to ascertain each group’s diet and feces methionine concentration. Briefly, the weighed samples were homogenized, followed by water and methanol addition. The mixture was then vortexed for extraction and centrifuged to obtain the supernatant late. Subsequently, the supernatant was swirled and shaken with water, internal standard, and isopropyl alcohol (1% formic acid) accession. The upper solution was centrifuged and combined with PBS in a 1.5 mL centrifuge tube, and the mixture introduced with the derivative reagent was derived at 50 °C for 10 min. Thereafter, the amino acid concentration was gauged by the column and diluted 10 times with water. Serum amino acid concentration was also measured using this method. The apparent digestibility of methionine was calculated using the following formula: nutrient apparent digestibility (%) = (the content of a nutrient in the diet − the content of the nutrient in the feces)/the content of the nutrient in the diet × 100.

### 2.4. Serum Biochemical

Blood samples (5 mL) were drawn from the vein, coagulated naturally for 30 min at room temperature, and then centrifuged for 10 min at 3000 r/min. The supernatant was gathered in a centrifuge tube and kept at −20 °C for follow-up usage. Afterward, 200 μL was taken into a 1.5 mL centrifuge tube to determine the quantities of serum ALT, AST, alkaline phosphatase (ALP), total protein (TP), albumin (ALB), glucose (Glu), total cholesterol (TCHO), and lactate dehydrogenase (LDH) using an automatic biochemical analyzer.

### 2.5. Skin Oxidative Stress Indicator

The skin tissue was sufficiently homogenized and centrifuged to take the supernatant. The protein content was quantified using the BCA method. The enzyme activity amplitudes of SOD, MDA, and ALT (Jiancheng, Nanjing, China), as well as the MSRA content (Enzyme Link, Shanghai, China) in the skin tissue were detected. Detailed procedures were executed following the kit instructions.

### 2.6. Oxidant Stress Index of Liver and Kidney

The supernatant was obtained after the liver and kidney tissues were fully homogenized and centrifuged. The protein concentration was monitored using the BCA assay, and the enzyme zing quotas of MDA, SOD, GPX, and CAT were measured in the liver and kidney tissue. The specific steps were performed according to the instructions provided in the kit.

### 2.7. RT-PCR

Rex rabbit liver and kidney RNA was extracted for fluorescence quantification using the previously described method [24]. Glyceraldehyde-3-phosphate dehydrogenase (GAPDH) was employed as a housekeeping gene, and the expression abundances were computed using the 2^−ΔΔCT^ method [25]; the list of primers is presented in Table 1.

### 2.8. Statistical Analysis

Data were subjected to a one-way ANOVA and Duncan’s multiple comparison analysis via the GLM procedure in SAS 9.4 (SAS Institute, Cary, NC, USA) statistical software. The results were presented as the mean ± standard error. The statistical significance threshold was set at *p* < 0.05, while 0.05 < *p* < 0.1 indicated a tendency in the statistical outcomes.

## 3. Results

### 3.1. Effects on Liver and Kidney Physiological Indicators

As indicated in Table 2, in contrast to the control group, heat stress notably decreased the liver weight and liver index of Rex rabbits (*p* < 0.05). The final body weight and kidney index tended to decrease, but the difference was non-significant. Additionally, 0.3% methionine supplementation increased liver weight and index (*p* < 0.05), but had no significant effect on final body weight and kidney index.

### 3.2. Slaughter Performance

**Figure 1:**
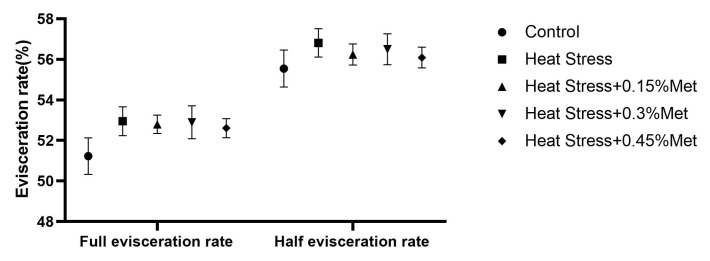
Effect of methionine on slaughter performance of heat-stressed Rex rabbits. Values shown are the mean ± standard error (*n* = 8). Values with different superscript letters are statistically significant (*p* < 0.05).

As depicted in Figure 1, there were no significant influences on the full and half evisceration rate of Rex rabbits in all experimental groups.

### 3.3. Methionine Concentration and Apparent Digestibility

As shown in Table 3, compared with the control group, the feces methionine contents of Rex rabbits were markedly accreted with the annexment of dietary methionine concentration of 0.45% (*p* < 0.05). Heat stress strikingly diminished the methionine apparent digestibility (*p* < 0.05), and 0.15% and 0.3% Met accretion significantly accelerated the methionine apparent digestibility in heat-stressed Rex rabbits (*p* < 0.05). Additionally, 0.45% Met supplementation significantly decreased methionine apparent digestibility (*p* < 0.05).

### 3.4. Other Amino Acid Concentrations in Serum

As demonstrated in Table 4, compared with the control group, during the heat stress group, glutamate (GLU) and aspartic acid (ASP) density in the serum were signally elevated (*p* < 0.05), methionine (Met) content was significantly decreased (*p* < 0.05), phenylalanine (PHE) concentration was increased, and TRP was decreased, but the differences were not significant. Additionally, 0.3% methionine supplementation significantly decreased GLU concentration (*p* < 0.05), and 0.15% and 0.3% methionine addition significantly increased TRP content (*p* < 0.05) but had no significant effect on ASP and PHE content.

### 3.5. Skin Oxidative Stress Assessment

As shown in Table 5, compared with the control group, heat stress prominently declined the dynamism of T-SOD and MRSA in the skin tissue of Rex rabbits (*p* < 0.05), yet it had no substantial impingement on the vril of MDA and ALT. In addition, 0.3% and 0.45% methionine supplementation significantly increased the skin MRSA content of heat-stressed Rex rabbits (*p* < 0.05), while 0.15% and 0.3% methionine addition tended to increase SOD level, but the difference was not significant.

### 3.6. Serum Biochemistry

**Figure 2:**
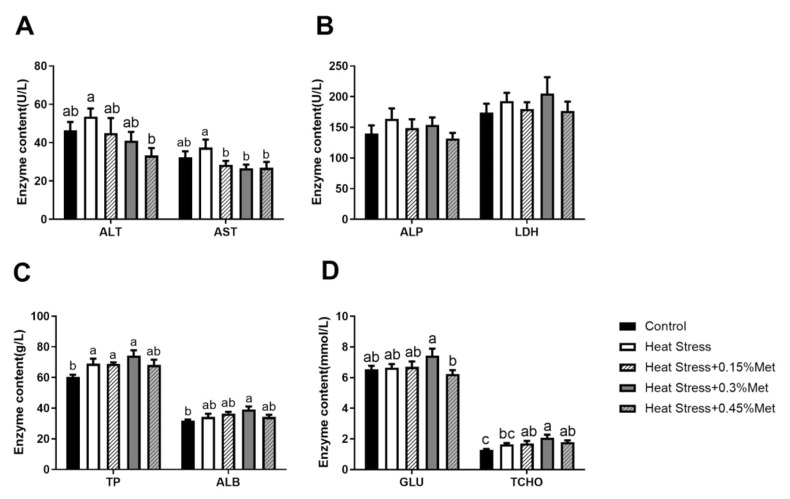
Effect of methionine on serum biochemistry of heat-stressed Rex rabbits. (**A**) ALT and AST concentrations; (**B**) ALP and LDH content; (**C**) TP and ALB concentrations; (**D**) GLU and TCHO content. Values shown are the mean ± standard error (*n* = 8). The same letters in the same graphics indicate insignificant differences (*p* > 0.05), while different letters indicate significant differences (*p* < 0.05).

As displayed in Figure 2, counterpointed to the control group, heat stress significantly enhanced the concentration of TP in serum (*p* < 0.05) and tended to increase the concentration of AST, but the difference was not significant (*p* < 0.1). There was no significant effect on ALP, LDH, ALB, and TCHO. Methionine supplementation significantly decreased AST concentration, and supplementation with 0.45% Met significantly decreased ALT concentration (*p* < 0.05).

### 3.7. Liver and Kidney Pathological Structure Diagnosis

**Figure 3:**
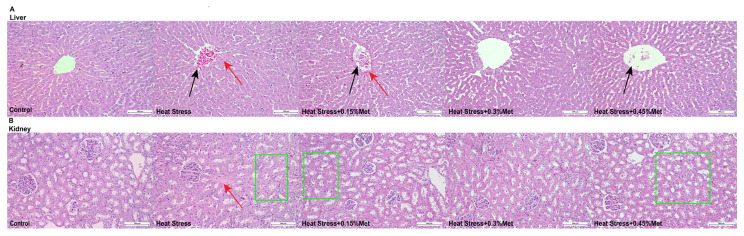
Effects of methionine on liver and kidney physiological structure in heat-stressed Rex rabbits. (**A**) Liver pathological section and (**B**) kidney pathological section. Black arrows indicate hyperchromatic nucleoplasm and nuclear fragmentation. Red arrows indicate fibrosis. Green boxes indicate vacuolization.

**Figure 4:**
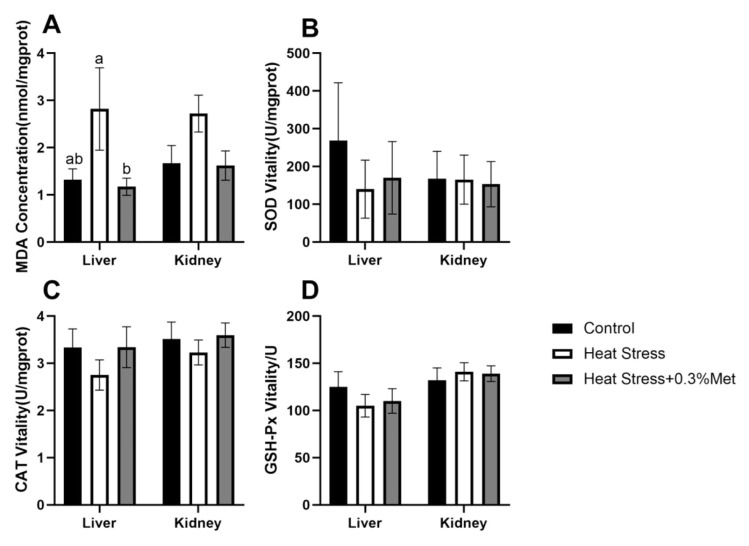
Effects of methionine on liver and kidney antioxidant enzyme activities in heat-stressed Rex rabbits. (**A**) MDA content; (**B**) SOD content; (**C**) CAT content; and (**D**) GSH-Px content. Values shown are the mean ± standard error (*n* = 8). The same letters in the same graphics indicate insignificant differences (*p* > 0.05), while different letters indicate significant differences (*p* < 0.05).

As Figure 3 specified, in the control group, the hepatocytes were compactly and seriatim arrayed in a cord-like configuration around the central vein and the normal sinusoid. In contrast, compared to the control group, the hepatocytes in the heat stress group were swollen and not neatly arranged. The central lobular veins were filled with a considerable number of red cells, featuring hyperchromatic nuclei or karyorrhexis (indicated by black arrows) and a slight degree of perivascular fibrosis (indicated by red arrows), which was alleviated by the Met affixion (Figure 4A). Compared with the control group, the heat stress group exhibited mild tubular fibrosis (red arrow), minor degenerative alterations in the renal tubular epithelium, and moderate vacuolization (enclosed by the green box), which were extenuated by the addition of different concentrations of Met (Figure 4B). To sum up, the 0.3% Met incorporation in the diet presented the most pronounced function in mitigating the oxidative functional harm to the liver and kidneys caused by heat stress. The heat stress + 0.3% Met group was chosen for further experiments.

### 3.8. Liver and Kidney Antioxidant Defense

As shown in Figure 4, Met supplementation significantly decreased MDA content in the liver of heat-stressed Rex rabbits (*p* < 0.05). However, the levels of SOD, CAT, and GPH-PX were not significantly affected.

### 3.9. Expression of Antioxidant Genes in the Liver and Kidney

**Figure 5:**
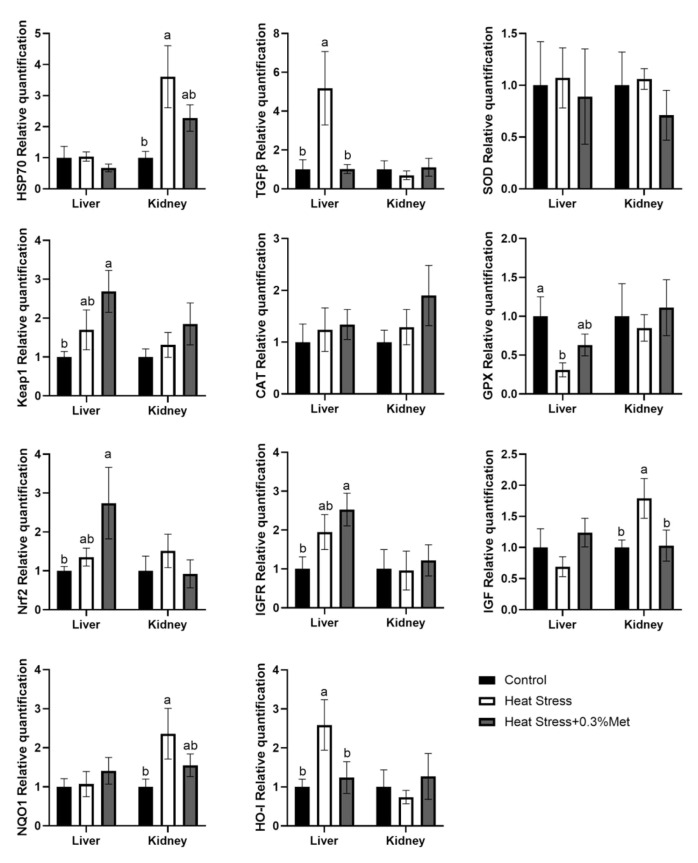
Effects of methionine on liver and kidney antioxidant genes in heat-stressed Rex rabbits. Values shown are the mean ± standard error (*n* = 8). The same letters in the same graphics indicate insignificant differences (*p* > 0.05), while different letters indicate significant differences (*p* < 0.05).

As displayed in Figure 5, in comparison with the control group, heat stress significantly accreted *TGFβ* and *HO-1* genes expression in the liver (*p* < 0.05), decreased *GPX* gene manifestation (*p* < 0.05), and tended to increase *keap1* and *Nrf2* gene expression, but the differences were not significant. Met significantly decreased *TGFβ* and *HO-1* gene expression (*p* < 0.05) and increased *keap1* and *Nrf2* gene expression to a certain extent, but statistical significance was not reached. Gene expressions of *HSP70*, *SOD*, *CAT*, *IGF* and *NQO1* were not significantly changed. In the kidney, compared with the control group, heat stress noticeably facilitated *HSP70*, *IGF*, and *NQO1* genes expression (*p* < 0.05), Met supplementation remarkably decreased *IGF* gene expression (*p* < 0.05), and to a certain extent slumped *HSP70* and *NQO1* genes expression, but the differences were not significant. There was no manifest impact on *TGFβ*, *SOD*, *Keap1*, *CAT*, *GPX*, *Nrf2*, *IGFR*, and *HO-1* gene amount.

## 4. Discussion

Fur acted as the first line of defense of Rex rabbits, safeguarding their bodies, skin, and internal organs from the external environment. Blood was the main source of nutrients for fur growth and maintenance. As the dominant metabolic organ of Rex rabbits, the liver, in addition to toxin removal, glycogen storage, protein synthesis, and defense against toxic chemicals, also stores blood, regulates the blood supply to the fur, harmonizes qi and blood, and nourishes the skin [27]. The kidneys are responsible for storing the essence and excreting metabolites, waste products, and toxins from the body while reabsorbing water and other serviceable substances [28]. Studies have shown that the skin response to heat stress parallels the kidney response [29], and many in vitro signs of kidney-related diseases also appear on the skin [30]. Oxidative stress caused by heat stress can affect Rex rabbits, resulting in a corresponding reduction in slaughter, carcass, and organ weight [31]. The experiment results also showed that heat stress decreased the final body weight, liver weight, and liver index and tended to decrease the kidney index. Methionine is an essential amino acid that plays important roles in protein synthesis, antioxidant defense, and methylation reactions [32]. By providing this substance, rabbits can better adapt to oxidative stress and maintain optimal liver and kidney function. The results showed that dietary supplementation of 0.3% methionine significantly increased liver weight, liver index, and apparent digestibility of methionine in heat-stressed Rex rabbits. These results indicated that 0.3% of methionine had protective effects on the liver and kidney of heat-stressed Rex rabbits. The amount of 0.45% Met may increase the metabolic burden on the liver and kidney, thereby reducing the apparent digestibility. Mammalian thermoregulation mechanisms between the sexes are highly conservative; sex differentiation mainly occurs in reproductive rather than basal metabolic levels [33]. Heat stress leads to oxidative damage by increasing reactive oxygen species (ROS) and malondialdehyde (MDA), while decreasing the activity of antioxidant enzymes (SOD, GSH-Px), and this effect is consistent in both male and female animals [34]. Therefore, this study does not consider the gender effect.

Under heat stress, the synthesis of animal protein in vivo and nitrogen deposition were reduced, while proteolysis increased [35]. The catabolism of amino acids was stimulated, thereby leading to all plasma-free amino acid concentrations being decreased, except for glutamate, aspartate, and phenylalanine [36]. The same outcomes were observed in the present experiment, indicating that heat stress promoted amino acid catabolism and methionine supplementation alleviated amino acid catabolism. The decomposed amino acids might enter the gluconeogenic pathway, consequently increasing glucose concentration in the serum.

The enzyme antioxidant system serves as the dominant defense scheme of the organism, counteracting oxidation. It is conducive to protecting cells from bruises caused by ROS. Oxidative stress occurs when there is an imbalance between ROS production and the ability of the antioxidant system to detoxify them [37]. MDA is a biomarker used to assess the degree of oxidative stress in organisms. Increased MDA activity indicates higher lipid peroxidation levels, impairing cell membranes [38,39]. T-SOD is a critical enzyme in the cellular antioxidant process, converting superoxide radicals into hydrogen peroxide and oxygen, thereby alleviating oxidative defect [40]. MSRA acts as a secondary antioxidant enzyme in protein repair [41]. MSRA was widely distributed in mammalian tissues, and its overexpression contributes to enhancing the organism’s ability to withstand oxidative stress and prolong its lifespan [42,43]. The findings of this study elucidated that heat stress perceptibly reduced T-SOD and MRSA protein levels in skin tissue, resulting in decreased antioxidant capacity. Methionine supplementation notably increased the concentration of MRSA and tended to strengthen T-SOD activity, slightly alleviating the oxidative stress induced by heat stress.

Aspartate aminotransferase (AST) and alanine aminotransferase (ALT) are key metabolic enzymes involved in amino acid metabolism and protein synthesis, and persistently elevated levels could indicate liver injury or dysfunction [44]. Serum ALT levels are a reliable indicator of liver trauma. Elevated ALT levels suggest hepatocyte destruction or necrosis. Increased serum AST concentration reflects the severity of liver injury, as AST is released into the bloodstream upon liver cell damage [45]. There was a tendency to increase serum ALT, AST, albumin (ALB), and total cholesterol (TCHO) levels under heat stress and increased (TP) concentration. Methionine supplementation significantly lowered ALT and AST levels, which exposed the extensive use of these antioxidants to safeguard tissues against oxidative wounding.

As an endogenous regulator of antioxidant capacity in the body, Nrf2 plays and increasingly important role in prolonging cellular REDOX homeostasis and accommodating inflammatory receptions in plenty of organs [46]. In response to oxidative stress, the interaction between Nrf2 and its inhibitor Keap1 was disrupted, and the liberated Nrf2 was translocated to the nucleus, where it bound to target genes like the detoxification enzymes NQO1, HO-1, and glutathione synthetase to gain the expression of several antioxidant genes (SOD and GSH-PX) [47]. In the liver, the increase in TGFβ suggests that heat stress may promote the risk of liver fibrosis by activating the Smad pathway [48]. In contrast, HO-1 can be activated by high temperature, which may reflect the mechanism of heme degradation to combat oxidative damage under heat stress conditions [49]. It is worth noting that although *Keap1* and *Nrf2* genes were upregulated, they contrasted with the significant activation of HO-1, suggesting that HO-1 expression may not be entirely dependent on the classical Keap1-Nrf2 pathway regulation but involve post-transcriptional modifications or regulation of non-coding RNA. Met intervention significantly reversed the abnormally high expression of TGFβ and HO-1. It slightly promoted the expression of *Keap1* and *Nrf2*, suggesting that Met may play a synergistic role in modulating the Keap1/Nrf2/HO1 antioxidant system by regulating DNA methylation or enhancing the activity of the thioredoxin system. However, the failure of Met to restore *GPX* expression may be related to its limited effect on selenium metabolism or selenocysteine synthesis. In the kidney, heat stress significantly activated *HSP70*, *IGF*, and *NQO1* expression, indicating that the kidney responds to heat injury through molecular chaperone-mediated protein repair (HSP70), growth factor signaling (IGF), and quinone detoxification (NQO1). There were no significant changes in *Keap1* and *Nrf2*, suggesting that NQO1 may be regulated by other transcription factors such as p53 [50]. Methionine addition may reduce metabolic load by blocking IGF receptor signaling and slightly downregulating *HSP70* and *NQO1*.

## 5. Conclusions

Heat stress can adversely affect Rex rabbits’ liver and kidney function and amino acid absorption ability, causing oxidative stress. Dietary methionine supplementation can reduce the liver and kidney oxidative damage caused by heat stress in Rex rabbits.

## Figures and Tables

**Table 1:** The primer sequences used for RT-PCR.

Gene	GenBank	Primer Sequences (5′→3′)	Product Size (bp)
*SOD*	XM_051854201.1	F: TTTCTGGACAAACCTGAGCCCTAAC R: CCGTCAGCCTCTCCTTGAACTTG	110
*CAT*	XM_002721425.3	F: CCAGTCTATTAGGTTCCATGTTCC R: CGATTATTGGCGTTTTGGTC	117
*GPx*	NM_001085444.1	F: CAGGAGAACGCCAAGAATGAGGAG R: GTTCACCTCGCACTTCTGGAAGAG	105
*NQO1*	XM_002711667.4	F: AGCGGCTCCATGTACTCTCTCC R: GAGTGTGCCCGATGCTGTATGTG	136
*HO-1*	XM_051846030.1	F: CCACCAAGTTCAAGCAGCTCTACC R: TTAGCCTCTTCCACCACCCTCTG	88
*Nrf2*	XM_051849401.1	F: AAGCAACTCAGCACCTTGTATCTGG R: GAATACATTGCCGTCCCTCGTCTG	114
*GAPDH*	NM_001082253.1	F: GGCTGCTTTTAACTCTGGCAAA R: CGTGGGTGGAATCATACTGGAA	101
*Keap1*	XM_008251549.3	F: CCTCAACCGCCTGCTCTATGC R: ATCCGCCACTCGTTCCTCTCC	96

Note: *TGFβ*, *HSP70*, *IGF1*, and *IGFR* primer sequences refer to the previous [26].

**Table 2:** Effect of methionine on liver and kidney index of heat-stressed Rex rabbits.

Items	Control	Heat Stress	Heat Stress +0.15% Met	Heat Stress +0.3% Met	Heat Stress +0.45% Met	R-MSE	*p*-Value
Initial weigh (kg)	1.97 ± 0.05	1.97 ± 0.04	1.96 ± 0.03	1.95 ± 0.04	1.93 ± 0.04	0.2220	0.9388
Final Weight (kg)	2.46 ± 0.05 ^a^	2.35 ± 0.04 ^ab^	2.37 ± 0.03 ^ab^	2.36 ± 0.04 ^ab^	2.28 ± 0.04 ^b^	0.2200	0.0546
Liver (g)	55 ± 1.1 ^a^	46 ± 1.3 ^c^	50 ± 1.2 ^bc^	51 ± 1.5 ^ab^	48 ± 1.8 ^bc^	3.929	0.0017
Liver index (g/kg)	23 ± 0.7 ^a^	21 ± 0.8 ^b^	22 ± 0.5 ^ab^	23 ± 0.5 ^a^	22 ± 0.9 ^ab^	1.929	0.0604
Kidney (g)	13 ± 0.6	11 ± 0.4	10 ± 0.5	11 ± 0.7	10 ± 0.4	1.497	0.0072
Renal index (g/kg)	5.4 ± 0.28 ^a^	4.9 ± 0.17 ^ab^	4.5 ± 0.22 ^b^	5.0 ± 0.29 ^ab^	4.6 ± 0.18 ^b^	0.6586	0.0696

Note: Data shown are the mean ± standard error (*n* = 8). In the same row, values with the same letter superscripts indicate no significant difference (*p* > 0.05), while different letters indicate significant differences (*p* < 0.05).

**Table 3:** Methionine concentration and apparent digestibility.

Items	Control	Heat Stress	Heat Stress +0.15% Met	Heat Stress +0.3% Met	Heat Stress +0.45% Met	R-MSE	*p*-Value
Feed (mg/g)	1.95 ± 0.06 ^d^	1.90 ± 0.02 ^d^	8.15 ± 0.09 ^c^	14.5 ± 0.15 ^b^	20.8 ± 0.25 ^a^	0.1922	<0.0001
Feces (μg/g)	12.5 ± 0.87 ^b^	16.9 ± 4.7 ^b^	13.1 ± 2.8 ^b^	21.9 ± 3.8 ^b^	475 ± 216 ^a^	167.2	0.024
Apparent digestibility (%)	99.2 ± 0.05 ^b^	98.7 ± 0.08 ^c^	99.8 ± 0.01 ^a^	99.9 ± 0.01 ^a^	96.7 ± 0.18 ^d^	0.2587	<0.0001

Note: Data shown are the mean ± standard error (*n* = 8). In the same row, values with the same letter superscripts indicate no significant difference (*p* > 0.05), while different letters indicate significant differences (*p* < 0.05).

**Table 4:** Effect of methionine on serum amino acid concentrations in heat-stressed Rex rabbits.

Items	Control	Heat Stress	Heat Stress +0.15% Met	Heat Stress +0.3% Met	Heat Stress +0.45% Met	R-MSE	*p*-Value
ASP (μmol/L)	0.20 ± 0.01 ^b^	0.29 ± 0.01 ^a^	0.28 ± 0.01 ^a^	0.25 ± 0.02 ^ab^	0.29 ± 0.01 ^ab^	0.030	0.029
GLU (μmol/L)	0.69 ± 0.04 ^b^	0.88 ± 0.04 ^a^	0.76 ± 0.01 ^ab^	0.70 ± 0.06 ^b^	0.73 ± 0.05 ^ab^	0.077	0.081
PHE (μmol/L)	0.62 ± 0.04 ^b^	0.75 ± 0.04 ^ab^	0.78 ± 0.02 ^a^	0.74 ± 0.03 ^ab^	0.75 ± 0.04 ^ab^	0.067	0.102
MET (μmol/L)	0.41 ± 0.03 ^b^	0.36 ± 0.01 ^c^	0.48 ± 0.01 ^a^	0.50 ± 0.01 ^a^	0.52 ± 0.01 ^a^	0.026	<0.0001
TRP (μmol/L)	0.46 ± 0.02 ^ab^	0.39 ± 0.02 ^b^	0.50 ± 0.02 ^a^	0.49 ± 0.05 ^a^	0.44 ± 0.01 ^ab^	0.043	0.065

Note: Data shown are the mean ± standard error (*n* = 8). In the same row, values with the same letter superscripts indicate no significant difference (*p* > 0.05), while different letters indicate significant differences (*p* < 0.05).

**Table 5:** Effect of methionine on antioxidation in skin of heat-stressed Rex rabbits.

Items	Control	Heat Stress	Heat Stress +0.15% Met	Heat Stress +0.3% Met	Heat Stress +0.45% Met	R-MSE	*p*-Value
MDA (nmol/mgprot)	5.95 ± 0.97	7.36 ± 0.49	7.61 ± 4.4	3.82 ± 0.92	7.24 ± 0.61	5.11	0.6851
ALT (U/gprot)	7.28 ± 1.49	8.42 ± 1.99	7.94 ± 2.48	7.98 ± 2.49	6.11 ± 1.83	4.913	0.9219
T-SOD (U/mgprot)	12.2 ± 0.26 ^a^	10.2 ± 0.66 ^b^	11.1 ± 0.09 ^ab^	11.4 ± 0.34 ^ab^	10.5 ± 0.44 ^b^	0.994	0.0192
MSRA (IU/L)	6.64 ± 0.20 ^a^	4.08 ± 0.13 ^c^	3.41 ± 0.50 ^c^	5.34 ± 0.21 ^b^	6.80 ± 0.63 ^a^	0.863	<0.0001

Note: Data shown are the mean ± standard error (*n* = 8). In the same row, values with the same letter superscripts indicate no significant difference (*p* > 0.05), while different letters indicate significant differences (*p* < 0.05).

## Data Availability

The data supporting this study’s findings are available upon request from the corresponding author.

## Abbreviations

The following abbreviations are used in this manuscript:

ALT	Alanine aminotransferase
AST	Aspartate aminotransferase
MDA	Malondialdehyde
MSRA	Methionine sulfoxide reductase A
SOD	Superoxide dismutase
CAT	Catalase
GPx	Glutathione peroxidase
HS	Heat stress
ALP	Alkaline phosphatase
TP	Total protein
ALB	Albumin
Glu	Glucose
TCHO	Total cholesterol
LDH	Lactate dehydrogenase
Met	Methionine

## Appendix A

**Table A1:** Basic diet content and nutritional level (air-dried basis).

Ingredients	Content (%)	Nutrient Levels	Content
Corn	6.00	DM (%)	87.45
Wheat middling	12.00	DE ^(2)^ (MJ/kg)	9.87
Wheat bran	15.00	CP (%)	17.04
Corn germ meal	24.00	EE (%)	3.8
Alfalfa powder	9.00	CF (%)	18.78
Soybean meal	8.00	Ash (%)	7.26
Soybean oil	1.00	Ca (%)	0.87
Beanstalk powder	21.00	P (%)	0.43
Premix ^(1)^	4.00	Lys (%)	0.59
Total	100.00	Met (%)	0.19

^(1)^ The premix provided the following per kg of diets: VA 8000 IU, VB_2_ 6 mg, VB_3_ 50 mg, VB_5_ 50 mg, VB_6_ 2 mg, VB_12_ 0.02mg, VB_7_ 0.2 mg, VD 900IU, VE 30 mg, VK 2 mg, Cu (as copper sulfate) 40 mg, Fe (as ferrous sulfate) 60 mg, Mn (as manganese sulfate) 10 mg, Zn 50mg, CaHPO_4_ 1500 mg, choline chloride 500 mg, Diclazuril 37.5 mg, Stone powder 750 mg, and NaCl 2500 mg. the rest is miscellaneous meal carrier complement. ^(2)^ Digestible energy (DE) was calculated theoretically, while other values were measured.
